# Crude Radix Aconiti Lateralis Preparata (Fuzi) with *Glycyrrhiza* Reduces Inflammation and Ventricular Remodeling in Mice through the TLR4/NF-*κ*B Pathway

**DOI:** 10.1155/2020/5270508

**Published:** 2020-10-20

**Authors:** Ping Yan, Wen Mao, Lushuai Jin, Mingsun Fang, Xia Liu, Jiali Lang, Lu Jin, Beibei Cao, Qiyang Shou, Huiying Fu

**Affiliations:** ^1^Department of Internal Medicine, University Hospital, Qufu Normal University, Qufu, China; ^2^School of Basic Medicine, Zhejiang Chinese Medical University, Hangzhou, China; ^3^The Second Clinical Medical College, Zhejiang Chinese Medical University, Hangzhou, China

## Abstract

Radix *Aconiti Lateralis Preparata* (Fuzi) is a traditional Chinese medicine. Its alkaloids are both cardiotonic and cardiotoxic; however, the underlying mechanisms are unclear. Compatibility testing and processing are the primary approaches used to reduce the toxicity of aconite preparations. The purpose of this study was to compare the effects of crude Fuzi (CFZ), CFZ combined with *Glycyrrhiza* (Gancao) (CFZ+GC), and prepared materials of CFZ (PFZ) on heart failure (HF) in C57BL/6J mice and explore the potential mechanisms of action of CFZ. Transverse aortic constriction (TAC) was used to generate the HF state, and CFZ (1.5 g·mL^−1^), PFZ (1.5 g·mL^−1^), or CFZ+GC (1.8 g·mL^−1^) was orally administered to the HF-induced mice daily. For the subsequent 8 weeks, hemodynamic indicators, ventricular pressure indices, and mass indices were evaluated, and histopathological imaging was performed. CFZ, CFZ+GC, and PFZ significantly improved left ventricular function and structure and reduced myocardial damage. CFZ+GC was more effective than CFZ and PFZ, whereas CFZ had higher toxicity than CFZ+GC and PFZ. CFZ and CFZ+GC attenuated ischemia-induced inflammatory responses and also inhibited Toll-like receptor-4 (TLR4) and nuclear factor kappa beta (NF-*κ*B) action in the heart. Moreover, mass spectrometry analysis revealed a decrease in the levels of toxic components of CFZ+GC, whereas those of the protective components were increased. This study suggested that GC reduces the toxicity and increases the efficacy of CFZ on HF induced by TAC. Furthermore, GC+CFZ reduces the risk of HF by ameliorating the inflammation response, which might be partially related to the inhibition of the TLR4/NF-*κ*B pathway.

## 1. Introduction

Heart failure (HF) is a clinical syndrome characterized by ventricular filling or ejection impairment that might be accompanied by signs of structural and functional cardiac abnormalities [1]. The main pathological mechanisms that lead to HF include increased hemodynamic overload, ventricular remodeling, ischemia-related dysfunction, neurohumoral stimulation, abnormal cardiac calcium circulation, and accelerated apoptosis [2, 3]. There are approximately 38 million patients with HF worldwide, and the number is increasing as the population ages [4]. Although medicine and device-based therapy improve survival in patients with HF, they have been unable to prevent this disease from progressing and cannot restore the quality of life for patients; the 5-year mortality rate remains at 50% [5]. Owing to the significant morbidity and mortality of HF, it has become a huge challenge in terms of socioeconomics and medical resources [6]. Therefore, it is important to develop effective treatments.

Traditional Chinese medicine (TCM) plays an indispensable role in the prevention and treatment of many diseases, including HF [7–9]. Almost all patients in China with HF are administered Chinese herbs or proprietary Chinese medicines combined with Western medicines because TCM can not only enhance heart function but also reduce the side effects of Western medicine [10, 11]. Radix *Aconiti Lateralis Preparata* (Fuzi), the lateral root of *Aconitum carmichaelii* Debx, has already been used in Asia for 2,000 years [12]. The alkaloids contained in Fuzi have widespread pharmacological activities, such as cardiotonic, antiarrhythmic, analgesic, anti-inflammatory, antiaging, immunomodulatory, and antitumor effects; however, they are also cardiotoxic [13], which leads to a conundrum regarding the use of Fuzi. Compatibility and processing are the primary methods to reduce the toxicity of Fuzi. For example, combination with *Glycyrrhizae Radix et Rhizoma* (Gancao, GC), another frequently used Chinese herb, attenuates the toxicity of Fuzi and is used as a compatible ingredient for Fuzi as a classic herb pair [14]. However, there are no modern standards for the use of crude Fuzi (CFZ), prepared materials of Fuzi (PFZ), and CFZ with a compatible ingredient because of the lack of in-depth research. This hinders the application of Fuzi in the treatment of HF in contemporary clinical settings.

In this study, a mouse HF model was used to investigate the effects of processing and compatibility on the pharmacological activity and toxicity of CFZ and explore the underlying mechanism. In addition, the composition of CFZ with enhanced activity and reduced toxicity was analyzed before and after compatibility to provide references for the proper use of CFZ in clinical practice.

## 2. Methods

### 2.1. Preparation of Extracts

After CFZ (purchased from Jiangyou Sichuan Zhongba Fuzi Technology Development Co., Ltd., Sichuan, China) extraction with 20x pure water three times, the extracts were combined and concentrated via rotary evaporation to 1.5 g·mL^−1^. PFZ (purchased from Jiangyou Sichuan Zhongba Fuzi Technology Development Co., Ltd., Sichuan, China) at 100 g was immersed 10 times in water for 30 min and boiled for 1 h. The supernatant was filtered and concentrated to 1.5 g·mL^−1^. CFZ (100 g) and GC (20 g; purchased from the TCM Clinic of Zhejiang University of Traditional Chinese Medicine, Hangzhou, China) were immersed in deionized water (1,200 mL, 1 : 10*w*/*v*) for 30 min and decocted by boiling for 1 h. The two liquids were combined and concentrated to 1.8 g·mL^−1^.

### 2.2. Identification of Extracts

Based on previous methodology [15], the components in the water extracts of CFZ and CFZ+GC were determined by ultrahigh performance liquid chromatography-quadrupole time-of-flight mass spectrometry (UPLC-Q/TOF MS). The chromatographic conditions were as follows: the chromatographic column was a ZORBAX Eclipse Plus C18 column (2.1 × 100 mm, 1.8 *μ*m, Agilent 1290-6545 UPLC-Q/TOF MS, Santa Clara, CA, USA); mobile phase: A solvent (0.1% formic acid solution), B solvent (0.1% acetonitrile); flow rate: 0.3 mL/min; elution procedure: 2 min, 95% A, 5% B; 20 min, 100% B; 25 min, 100% B; column temperature: 40°C; sample and standard sample injection volume: 2 *μ*L.

The mass spectrometry experimental conditions were as follows: ion source mode: electrospray ionization, negative ion mode; drying gas temperature: 325°C; drying gas flow rate: 1012 L/min; sheath gas temperature: 370°C; sheath gas flow rate: 12 L/min; nebulizer pressure: 35 psi; capillary voltage: 3000 V; nozzle voltage: 1500 V; acquisition mode: full scan, *m*/*z*: 100–1700.

### 2.3. Experimental Groups

All animal protocols were approved by the Zhejiang Chinese Medical University Laboratory Animal Research Center, and the experiments were performed in compliance with the “Guide for the Care and Use of Laboratory Animals” from the US National Institute of Health. We used 50 healthy, male, 10-week-old, clean CFZ-grade C57BL/6J mice (purchased from Shanghai Lingchang Biotechnology Co., Ltd., Shanghai, China) that weighed 22 ± 2 g. The mice were randomly divided into five experimental groups as follows: (1) sham operation group (*n* = 7), (2) model group (*n* = 10), (3) CFZ group (*n* = 10, CFZ 1.5 g·mL^−1^), (4) CFZ+GC group (*n* = 10, CFZ+GC 1.8 g·mL^−1^), and (5) PFZ group (*n* = 10, PFZ 1.5 g·mL^−1^). HF was induced in all groups, except the sham group, by transverse aortic constriction (TAC). After 4 weeks, the sham and model groups were administered distilled water intragastrically and the treatment groups were administered different treatments for 8 weeks.

### 2.4. Establishment of a Mouse Model of HF Induced by TAC

Based on a previous study [16], the mice were anesthetized with 0.3% sodium pentobarbital (75 mg·kg^−1^) intraperitoneally and ophthalmic scissors were used to make a longitudinal incision of 0.5–1 cm at the midline of the neck and chest of the mouse. After the separation of the two thymus halves using microforceps, the aortic arch and two carotid arteries were observed. Microtweezers were used to pass a 6-0 nylon suture gently between the left common carotid artery and the right innominate artery. A homemade L-shaped 26G cushion needle was used to ligate the aortic arch, and the cushion needle was carefully removed after ligation. A significant increase in the right common carotid artery pulsation was observed after successful aortic arch stenosis. Penicillin sodium (5000 U/head) was injected intramuscularly 3 days after the operation.

### 2.5. Measurement of Hemodynamic Indicators and Ventricular Pressure Indicators

Eight weeks after dosing, we performed an ultrasound examination of mouse hearts to obtain M-mode echocardiographic images using a Vevo 1100 ultra-high-resolution small animal ultrasound imaging system and an MS400 ultrasound probe (VisualSonics, Toronto, Canada). The images were analyzed to measure the hemodynamic indicators. Three consecutive cardiac cycles were analyzed, and the average value was used to calculate ventricular pressure indicators.

### 2.6. Measurement of Mass Indices

Mass indices were measured using the methods reported in a previous study [16]. Having measured hemodynamic parameters after 8 weeks, the mice were sacrificed by placing them in a sealed chamber containing CO_2_. Briefly, the time expected for mice to lose consciousness is typically within 2 to 3 min. Each mouse was observed for a lack of respiration and faded eye color, and the flow of CO_2_ was maintained for a minimum of 1 min after respiration had ceased. If both signs were observed, the mouse was removed from the cage. After euthanasia, the chest was rapidly opened to remove the heart, thymus, and spleen to calculate the weight index.

### 2.7. Histopathological Examination

The heart was cut transversely into two parts from the median plane. The apical part was fixed with neutral formaldehyde and conventional paraffin embedding, and hematoxylin and eosin (HE) staining was performed. The pathological sections were scanned using the NanoZoomer 2.0 RS digital slice scanning device to observe the pathological changes in the heart.

### 2.8. Detection of Serum Inflammatory Markers

From each mouse, 1 mL of blood was extracted from the abdominal aorta and stored at room temperature for 30 min. Then, the sample was centrifuged at 3,500 rpm for 10 min and the supernatant was used to detect the levels of inflammatory factors, tumor necrosis factor- (TNF-) *α*, interleukin- (IL-) 6, interferon- (IFN-) *γ*, and monocyte chemoattractant protein-1 (MCP-1), in serum. BD™ Cytometric Bead Array mouse inflammation kits were used according to the manufacturer's instructions (BD Biosciences, San Jose, CA, USA).

### 2.9. Quantitative RT-PCR (qRT-PCR) Assay to Monitor the Gene Expression Levels of Inflammatory Factor, Chemokine, Toll-Like Receptor (TLR) 2, and TLR4 Genes

Total RNA was extracted from heart tissue using Trizol® total RNA isolation reagent (Shanghai Yubo Biotechnology Co., Ltd., Shanghai, China) according to the manufacturer's protocol and reverse transcribed to cDNA. Quantitative PCR was performed in duplicate using a TB Green Master Mix (Takara Bio Co., Ltd., Beijing, China) and a Bio-Rad C1000 thermal cycler. Genes were amplified using the following primer pairs (Sangon Biotech, Shanghai, China) (Table 1); the expression of *β*-actin was simultaneously analyzed as the endogenous control (Table 1).

### 2.10. Western Blotting Analysis of Nuclear Factor-Kappa Beta (NF-*κ*B)

Total protein was extracted from heart tissue using a radioimmunoprecipitation assay buffer (Biyuntian Biotechnology Co., Ltd., Shanghai, China) containing phenylmethanesulfonyl fluoride. The extract was homogenized (Bertin Technologies, France) and lysed on ice for 15 min. Then, the sample was centrifuged at 12,000 rpm for 10 min and the supernatant was used to determine the total protein in the heart tissue. Protein concentrations were determined using a bicinchoninic acid protein-determination kit (Biyuntian Biotechnology Co., Ltd.). Protein samples were separated using 10% sodium dodecyl sulfate gel electrophoresis, transferred onto polyvinylidene fluoride membranes, and blocked for 2 h with a solution containing 5% skim milk powder (Sangon Biotech, Shanghai, China) in Tris buffer. The membrane was incubated overnight at 4°C with primary antibodies for NF-*κ*B, TLR4, and *β*-actin (Cell Signaling Technology, Beverly, MA, USA), which were diluted at the concentration recommended by the manufacturer. Then, the membrane was incubated with horseradish peroxidase-labeled secondary antibody in Tris-buffered saline containing 5% skim milk at room temperature for 2 h. Proteins were visualized using an ultrasensitive chemiluminescence imaging system (Jingtan Biotechnology (Shanghai) Co., Ltd., Shanghai, China), and the AlphaView FluorChem FC3 3.4.0.0 software (ProteinSimple, San Jose, CA, USA) was used for the quantitative analysis of the ribbons and normalized to *β*-actin.

### 2.11. Statistical Methods

SPSS 26.0 was used for statistical analysis. Quantitative data are presented as the mean ± standard deviation. ANOVA was used for the comparison of normal distribution data, and a nonparametric test was used for the comparison of nonnormal distribution continuous data. A chi-square test was used to compare survival time. *P* < 0.05 was considered statistically significant.

## 3. Results

### 3.1. CFZ+GC and PFZ Increased Survival Rate of Mice with HF

**Figure 1:**
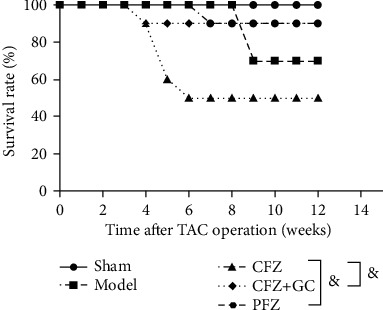
Survival rate of heart failure (HF) with different treatments. ^&^*P* < 0.05 versus the crude Fuzi (CFZ) group.

The survival rate was lower in the model group than that in the sham group (from 100% to 70%) with the progression of HF (Figure 1). However, CFZ treatment decreased the survival rate of mice to 50% lower than that in the model group, but in the CFZ+GC and PFZ treatment group, the survival rate was significantly higher than that in the CFZ group (from 50% to 90%) (*P* < 0.05).

### 3.2. CFZ+GC Improved Hemodynamic Indicators of Mice with HF

**Figure 2:**
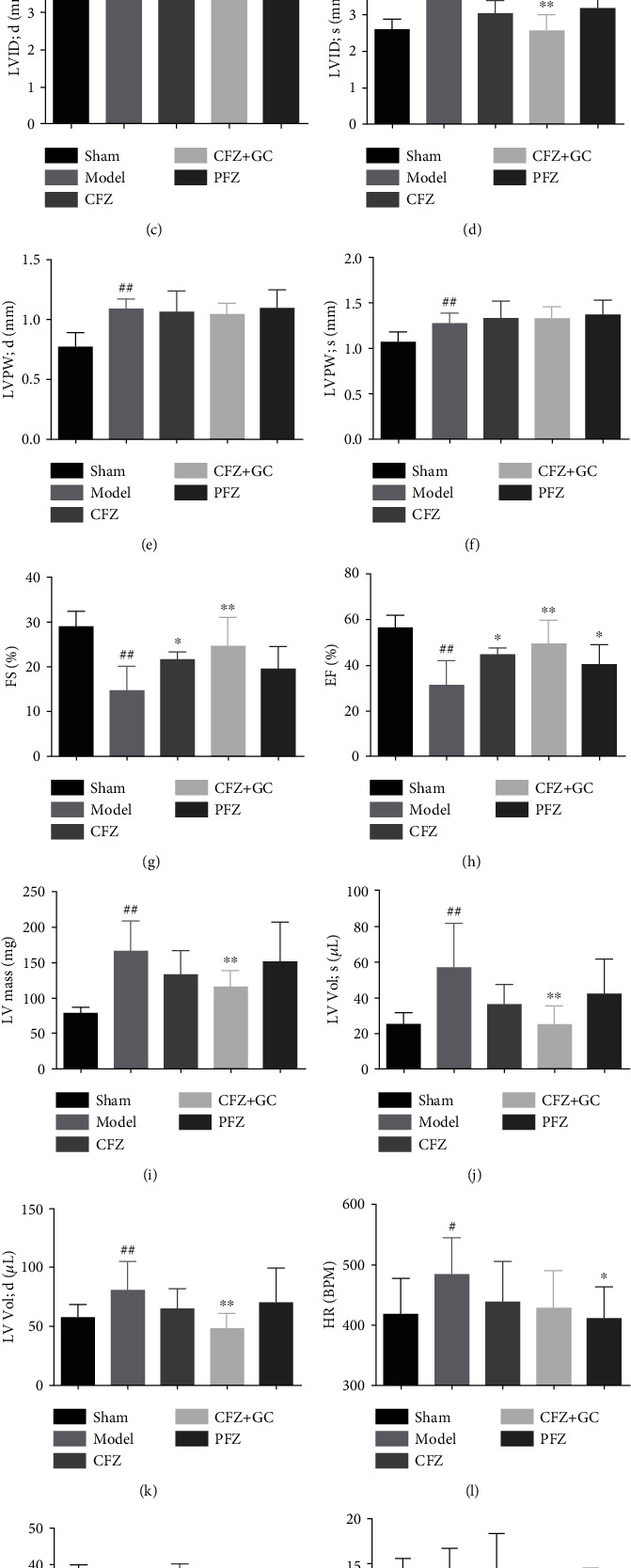
Hemodynamic indicators in mice of each group: (a) IVS; d: left ventricular end-diastolic anterior wall thickness; (b) IVS; s: left ventricular end-systolic anterior wall thickness; (c) LVID; d: left ventricular end-diastolic diameter; (d) LVID; s: left ventricular end-systolic diameter; (e) LVPW; d: left ventricular end-diastolic wall thickness; (f) LVPW; s: left ventricular end-systolic posterior wall thickness; (g) EF: ejection fraction; (h) LV mass corrected: left ventricular-corrected weight; (i) FS: fractional shortening; (j) HR: heart rate; (k) SV: stroke volume; (l) LV Vol; s: left ventricular end-systolic volume; (m) LV Vold; d: left ventricular end-diastolic volume; (n) CO: cardiac output. Sham group (*n* = 7), model group (*n* = 7), crude Fuzi (CFZ) group (*n* = 5), CFZ combined with *Glycyrrhiza* (CFZ+GC) group (*n* = 9), and prepared materials of Fuzi (PFZ) group (*n* = 9). All data are presented as the mean ± SD. ^#^*P* < 0.05 and ^##^*P* < 0.01 versus model group.; ^∗^*P* < 0.05 and ^∗∗^*P* < 0.01 versus model group.

Most hemodynamic indicators, except stroke volume and cardiac output, were significantly changed in mice subjected to TAC compared with the sham group based on the cardiac ultrasound (*P* < 0.05; Figure 2). In the CFZ+GC treatment group, the left ventricular end-diastolic diameter (IVS; d), left ventricular end-systolic diameter (IVS; s), left ventricular end-diastolic volume (LV Vold; d), left ventricular end-systolic volume (LV Vol; s), and left ventricular-corrected weight (LV mass corrected) were significantly lower and the fractional shortening (FS) and ejection fraction (EF) were significantly higher than those in the model group (*P* < 0.01). In addition, the indicators of EF and FS in the CFZ and PFZ groups were both noticeably higher than those in the model group (*P* < 0.05). The hemodynamic indicators with CFZ+GC treatment were improved more than those with the other treatments.

### 3.3. CFZ, CFZ+GC, and PFZ Improved Ventricular Pressure Indicators in Mice with HF

**Figure 3:**
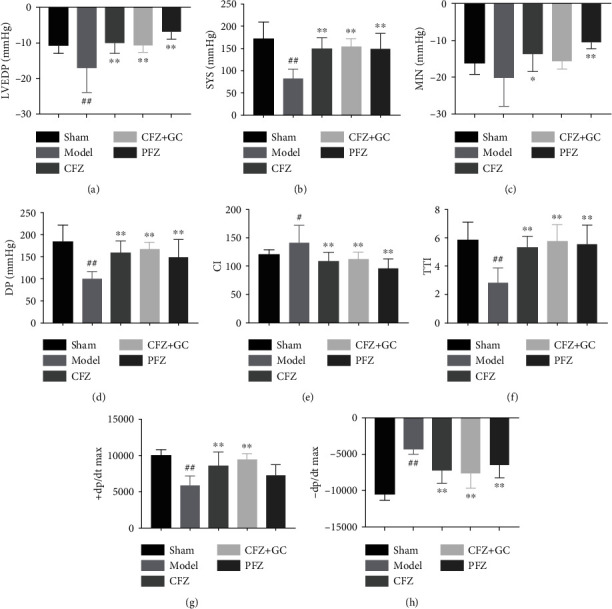
Ventricular pressure indicators in mice of each group: (a) LVEDP: left ventricular end-diastolic pressure; (b) SYS: systolic pressure; (c) MIN: minimum intracardiac pressure; (d) DP: diastolic pressure; (e) CI: cardiac index; (f) TTI: tension time index; (g) +dp/dt max: maximum rate of increase of left ventricular pressure; (h) −dp/dt max: maximum rate of left ventricular pressure decline. Sham group (*n* = 7), model group (*n* = 7), crude Fuzi (CFZ) group (*n* = 5), CFZ combined with *Glycyrrhiza* (CFZ+GC) group (*n* = 9), and prepared materials of Fuzi (PFZ) group (*n* = 9). All data are presented as the mean ± SD. ^#^*P* < 0.05 and ^##^*P* < 0.01 versus the sham group; ^∗^*P* < 0.05 and ^∗∗^*P* < 0.01 versus the model group.

Most ventricular pressure indicators were significantly different between the model group and the sham group (*P* < 0.05 or *P* < 0.01; Figure 3). However, the indices of left ventricular end-diastolic pressure (LVEDP), systolic pressure (SYS), minimum intracardiac pressure (MIN), diastolic pressure (DP), cardiac index (CI), and tension time index (TTI) in all treatment groups were significantly lower (*P* < 0.01), whereas the maximum rate of the left ventricular pressure decline indicator (−dp/dt max) was significantly higher (*P* < 0.01) than that in the model group. The maximum rate of increase of left ventricular pressure (+dp/dt max) was significantly higher in the CFZ and CFZ+GC groups (*P* < 0.01) than that in the model group, whereas the PFZ group did not show a statistically significant difference.

### 3.4. CFZ, CFZ+GC, and PFZ Improved Mass Indices of Mice with HF

**Figure 4:**
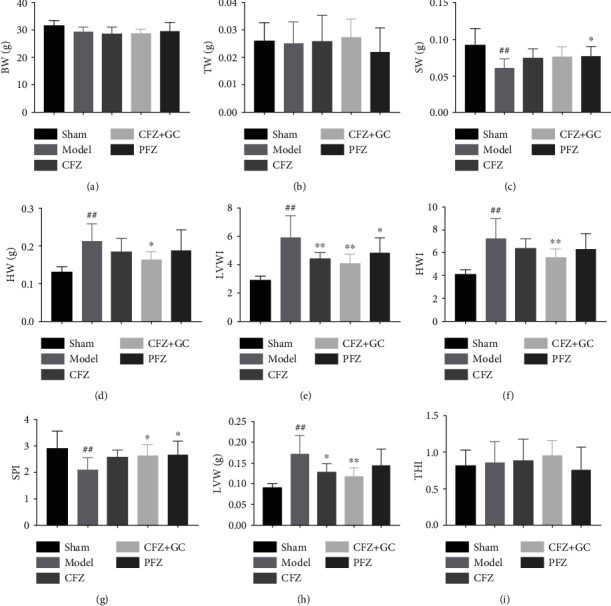
Mass indices in mice of each group: (a) BW: body weight; (b) TW: thymus weight; (c) SW: spleen weight; (d) HW: heart weight; (e) LVW: left ventricle weight; (f) THI: thymus weight index; (g) SPI: spleen weight index; (h) HWI: heart weight index; (i) LVWI: left ventricle weight index. Sham group (*n* = 7), model group (*n* = 7), crude Fuzi (CFZ) group (*n* = 5), CFZ combined with *Glycyrrhiza* (CFZ+GC) group (*n* = 9), and prepared materials of Fuzi (PFZ) group (*n* = 9). All data are presented as the mean ± SD. ^##^*P* < 0.01 versus the sham group; ^∗^*P* < 0.05 and ^∗∗^*P* < 0.01 versus the model group.

Spleen weight (SW) and spleen weight index (SPI) were significantly lower (*P* < 0.01), and heart weight (HW), left ventricle weight (LVW), heart weight index (HWI), and left ventricle weight index (LVWI) were significantly higher in the model group than those in the sham group (*P* < 0.01; Figure 4). However, LVW, LVWI, and HWI in the CFZ+GC group (*P* < 0.01), LVW, SW, and LVWI in the PFZ group (*P* < 0.05), and LVW and LVWI in the CFZ group (*P* < 0.05) were significantly lower than those in the model group.

### 3.5. CFZ, CFZ+GC, and PFZ Improved Histopathological Results of Mice with HF

**Figure 5:**
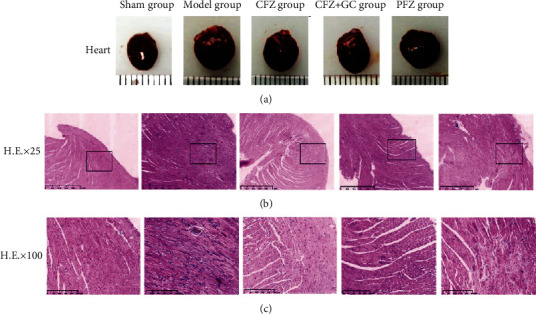
Heart morphologies and myocardial hematoxylin and eosin (HE) staining in each group. (a) Representative images of the heart. (b) Representative images for the pathological changes of the heart, scale bar = 1 mm. (c) Representative images for the pathological changes of the heart, scale bar = 250 *μ*m.

The heart volume in the model group was significantly higher than that in the sham group, and treatments with CFZ, CFZ+GC, and PFZ reduced cardiac hypertrophy to varying degrees (Figure 5(a)). The HE staining showed that the myocardial fibers were arranged neatly and there was no interstitial edema or myocardial fiber rupture in the sham group, whereas the histopathological structure of the heart in the model group had widened and broken myocardial tissue gaps, vascular degeneration, edema, necrosis, atrophy, and other pathological changes in myocardial cells. However, these pathological changes were alleviated to varying degrees in the treatment, especially the CFZ+GC, groups, in which the cardiac pathology of mice was notably improved compared with that of the model group mice (Figures 5(b) and 5(c)).

### 3.6. CFZ, CFZ+GC, and PFZ Inhibited Inflammation and Chemokine Levels of Mice with HF

**Figure 6:**
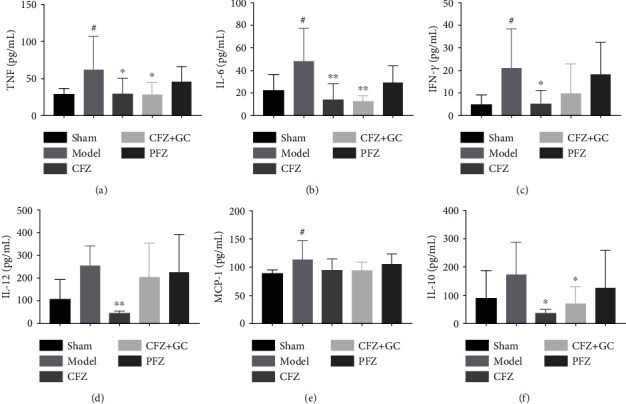
Inflammation and chemokine levels in mice of each group: (a–f) tumor necrosis factor- (TNF-) *α*, interleukin- (IL-) 6, interferon- (IFN-) *γ*, IL-12, and monocyte chemoattractant protein- (MCP-) 1 levels, respectively. All data are presented as mean ± SD (*n* ≥ 5/group). ^#^*P* < 0.05 and ^##^*P* < 0.01 versus the sham group; ^∗^*P* < 0.05 and ^∗∗^*P* < 0.01 versus the model group.

The protein levels of TNF-*α*, IL-6, IFN-*γ*, and MCP-1 in the serum of mice in the HF groups were higher than those in the sham group (*P* < 0.05; Figure 6). IL-12 and IL-10 levels were also higher, but this was not statistically significant (*P* > 0.05). CFZ treatment had significantly lower TNF-*α*, IL-6, IFN-*γ*, IL-12, and IL-10 levels than the other HF groups (*P* < 0.05 or *P* < 0.01). However, only TNF-*α*, IL-6, and IL-10 levels in the CFZ+GC treatment group were significantly lower than those in the other HF groups (*P* < 0.05 or *P* < 0.01). There was no significant difference in protein levels between PFZ treatment and any other group (*P* < 0.05) (Figure 6).

**Figure 7:**
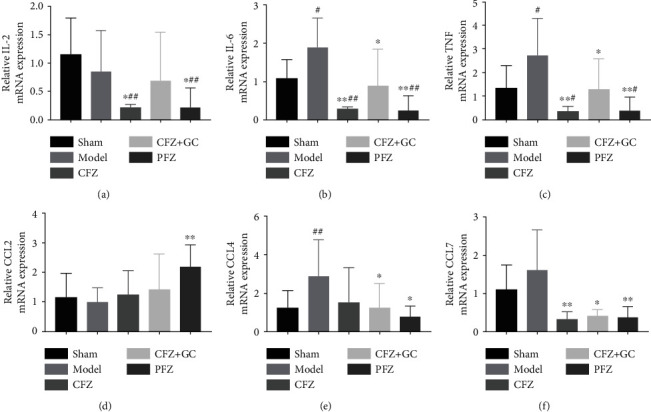
The mRNA expression of inflammatory factors and chemokines in heart of each group: (a–f) inflammation factors interleukin- (IL-) 2, tumor necrosis factor- (TNF-) *α*, IL-6, and chemokine ligand (CCL) 2, CCL4, and CCL7 mRNA levels, respectively. All data are presented as mean ± SD (*n* ≥ 5/group). ^#^*P* < 0.05 and ^##^*P* < 0.01 versus the sham group; ^∗^*P* < 0.05 and ^∗∗^*P* < 0.01 versus the model group.

In addition, the expression of inflammation factors, TNF-*α*, IL-6, and chemokine ligand (CCL) 4 mRNA, in the heart tissue of mice in the HF groups, was higher than that in the sham group (*P* < 0.05 or *P* < 0.01; Figures 7(b), 7(c), and 7(e)). In the CFZ, CFZ+GC, and PFZ groups, the mRNA levels of IL-2, TNF-*α*, IL-6, and CCL7 were significantly lower than those in the model group (*P* < 0.05 or *P* < 0.01; Figures 7(a)–7(c) and 7(f)). In the CFZ+GC and PFZ groups, the mRNA level of CCL4 was significantly lower than that in the model group (*P* < 0.05; Figure 7(d)). However, the mRNA levels of IL-2, TNF-*α*, and IL-6 in the CFZ group were significantly lower than those in the sham group (*P* < 0.05 or *P* < 0.01; Figures 7(a)–7(c)).

### 3.7. CFZ, CFZ+GC, and PFZ Improved the Inflammation Pathway in Mice with HF

**Figure 8:**
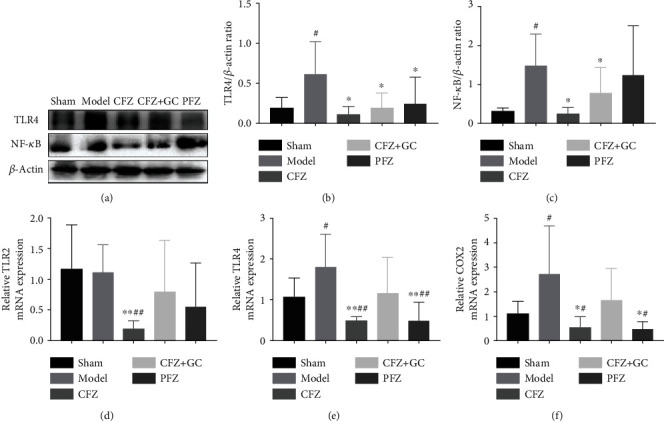
Toll-like receptor-4 (TLR4)/nuclear factor kappa beta (NF-*κ*B) pathway detection in the heart among mice in all groups. (a) Representative images for TLR4 and NF-*κ*B protein detection by western blotting. (b–c) TLR4 and NF-*κ*B protein expression in the heart of mice, respectively. (d–f) mRNA expression of TLR2, TLR4, and cyclooxygenase-2 (COX2) in the heart of mice, respectively. ^#^*P* < 0.05 and ^##^*P* < 0.01 versus the sham group; ^∗^*P* < 0.05 and ^∗∗^*P* < 0.01 versus the model group.

TLR4 and NF-*κ*B signaling pathways were involved in the inflammatory response to TAC-induced HF (Figure 8). HF significantly upregulated the protein expression of TLR4 and NF-*κ*B compared with the sham group, whereas CFZ, CFZ+GC, and PFZ downregulated the expression of these proteins.

In addition, the mRNA expression of TLR4 and cyclooxygenase-2 (COX-2) in heart tissues significantly increased after TAC induction, whereas CFZ and PFZ treatment resulted in a significant decrease in their expression, to lower than that in the sham group. Moreover, CFZ had a significant inhibitory effect on the expression of TLR2.

### 3.8. Analysis of CFZ and CFZ+GC Water Extracts by UPLC-Q/TOF MS

**Figure 9:**
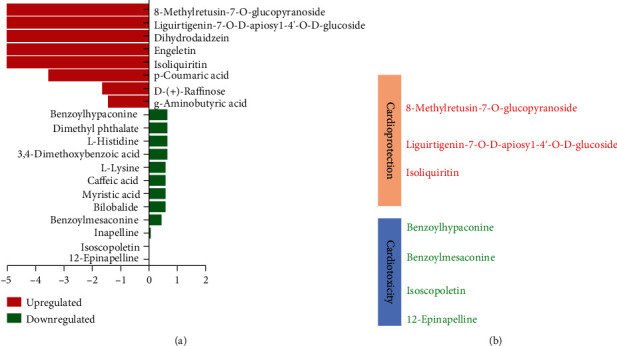
Component comparison of crude Fuzi (CFZ) and CFZ combined with *Glycyrrhiza* (CFZ+GC) related to function. (a) Significantly changed composition in CFZ+GC water extract compared with CFZ water extract. (b) Effects of changed ingredients on the heart.

Forty-seven components in the CFZ water extract (SI Table 1) and 49 components in the CFZ+GC water extract (SI Table 2) were identified. Comparing the composition of the two groups, there were some differences between the active components (Figure 9(a)). For example, the peak area of benzoylmesaconine and benzoylhypaconine, which considered the key toxic components of CFZ [17, 18], was reduced by half and isoscopoletin, detected in CFZ, was not detected in CFZ+GC, whereas some of the heart-protective components of GC, such as isoliquiritin, were detected (Figure 9(b)).

## 4. Discussion

HF is a complex clinical syndrome and represents the final stage of numerous heart diseases. This study showed that Fuzi significantly inhibited TAC-induced HF, improved the pathological changes of myocardial structure and cardiac function, and reduced the mortality rate when combined with GC, which suggested that the combination of CFZ and GC has great potential in the development of a treatment for HF in the future.

The +dp/dt max is highly sensitive to variable force factors and reflects the maximum shortening rate of myocardial contractile components. Therefore, +dp/dt max is a good indicator of the acute changes in contractile performance. The −dp/dt max reflects the maximum rate of myocardial fiber elongation during myocardial diastole [19, 20]. Both are common indicators to evaluate myocardial contraction and diastolic performance. CFZ, CFZ+GC, and PFZ groups all significantly improved these indicators, as well as other ventricular pressure indicators, such as LVEDP, SYS, MIN, DP, CI, and TTI. In addition, the CFZ+GC group partly improved the hemodynamic indicators and mass indices of mice with HF. Therefore, GC not only reduced the toxicity of CFZ but also increased the effects of CFZ on HF.

HF is associated with systemic inflammation [21–23]. The hemodynamic stress of HF induces sterile inflammation, and the resulting increased wall tension and mechanical stretch trigger the release of a range of proinflammatory cytokines, including TNF-*α*, IL-6, and IL-1*β*, as shown in our results. The TNF-*α* signaling pathway is associated with impaired systolic and diastolic function and poor cardiac remodeling [24]. However, clinical evidence suggests that the inhibition of TNF-*α* does not improve HF, rather increases the hospitalization rate for HF [25], suggesting the double-edged sword effect of TNF-*α* signaling. CFZ and CFZ+GC significantly reduced the serum levels of inflammatory factors such as TNF-*α* and IL-6. In addition, CFZ, CFZ+GC, and PFZ also can significantly reduce the mRNA levels of macrophage-related chemokines CCL7 and CCL4 in heart tissue [26, 27]. However, the mRNA levels of cardiac inflammatory factors in the CFZ group were significantly lower than those in the sham group, but not those in the CFZ+GC group. These showed CFZ has a strong inhibitory effect on inflammation.

TLR4 is most expressed in the heart and is involved in myocardial inflammation. TLR4 activation increases the production of IL-6 and TNF-*α*. NF-*κ*B is a transcriptional regulator that is activated by TNF-*α* and mediates inflammation, apoptosis, and extracellular matrix remodeling. However, NF-*κ*B has also been reported to have cardioprotective effects, such as reducing mitochondrial dysfunction and mitochondrial autophagy, inhibiting cell death, and inducing antioxidant effects [24]. CFZ significantly inhibited the mRNA levels of TLR4 and COX2 compared to those in the sham group. However, CFZ+GC treatment had no significant effect on mRNA expression in relation to inflammatory signaling pathways. This beneficial effect of NF-*κ*B may be lost owing to excessive inhibition of TNF-*α*, leading to progressive HF and death. Therefore, the toxicity of CFZ may be owing to a high-intensity blockade of TNF-*α*, which contributes to HF.

The clinical symptoms of Fuzi poisoning include dizziness, vomiting, hypotension, arrhythmia, chest tightness, palpitations, dyspnea, and coma [28]. In this study, we observed that GC significantly reduced the mortality associated with CFZ. In addition, the content of alkaloids, including monoester alkaloids of benzoylmesaconine and benzoylhypaconine that are associated with cardiotoxicity [17, 18] in CFZ+GC is significantly lower than that in CFZ alone. Moreover, the GC in the CFZ+GC group added some heart-protective components, such as flavonoids [29]. The changes in these components indicated that GC can alleviate the toxicity of CFZ and increase the protective effect of Fuzi on the heart.

## 5. Conclusions

CFZ, CFZ+GC, and PFZ exert protective effects on the heart by inhibiting the inflammatory response through the TLR4/NF-*κ*B pathway. However, CFZ is more toxic and PFZ has less efficacy than CFZ+GC. The combination of CFZ and GC is more effective for the treatment of HF in mice than CFZ or PFZ alone.

## Figures and Tables

**Table 1:** The primer sequences used in this study.

Gene^1^	Primer sequence	
	Forward primer (5′-3′)	Reverse primer (5′-3′)
CCL2	GATCCCAATGAGTAGGCTGG	CGGGTCAACTTCACATTCAAAG
CCL4	AAACCTAACCCCGAGCAAC	CTGTCTGCCTCTTTTGGTCA
CCL7	TCTCTCACTCTCTTTCTCCACC	GGTGATCCTTCTGTAGCTCTTG
TLR4	ATGGCATGGCTTACACCACC	GAGGCCAATTTTGTCTCCACA
TLR2	CTCTTCAGCAAACGCTGTTCT	GGCGTCTCCCTCTATTGTATTG
Cox2	TGCATTCTTTGCCCAGCACT	AAAGGCGCAGTTTACGCTGT
TNF-*α*	CAGGCGGTGCCTATGTCTC	CGATCACCCCGAAGTTCAGTAG
IL-6	TGAGCAGGATGGAGAATTACAGG	GTCCAAGTTCATCTTCTAGGCAC
IL-2	TGAGCAGGATGGAGAATTACAGG	GTCCAAGTTCATCTTCTAGGCAC
*β*-Actin	GTGACGTTGACATCCGTAAAGA	GCCGGACTCATCGTACTCC

## Data Availability

The data used to support the findings of the study is available in the supplementary materials.

## Abbreviations

BW: Body weight
CFZ: Crude Fuzi
CFZ+GC: Crude Fuzi with Glycyrrhiza
+dp/dt max: Maximum rate of increase of left ventricular pressure
EF: Ejection fraction
FS: Fractional shortening
HF: Heart failure
HR: Heart rate
HW: Heart weight
HWI: Heart weight index
IVS; d: Left ventricular end-diastolic anterior wall thickness
IVS; s: Left ventricular end-systolic anterior wall thickness
LVPW; d: Left ventricular end-diastolic wall thickness
LVPW; s: Left ventricular end-systolic posterior wall thickness
LVW: Left ventricle weight
LVWI: Left ventricle weight index
PFZ: Prepared materials of Fuzi
SPI: Spleen weight index
SW: Spleen weight
THI: Thymus weight index
TW: Thymus weight.

## Supplementary Materials

Supplementary materials SI Table 1: chemical information for identified compounds of CFZ. SI Table 2: chemical information for identified compounds of CFZ+GC.
