# Supplementary material for: Crude Radix Aconiti Lateralis Preparata (Fuzi) with Glycyrrhiza Reduces Inflammation and Ventricular Remodeling in Mice through the TLR4/NF-κB Pathway
Source: Mediators Inflamm. 2020 Oct 20;2020:5270508. doi: 10.1155/2020/5270508 (PMC7593747; doi:10.1155/2020/5270508)
Supplement: Supplementary materials — SI Table 1: chemical information for identified compounds of CFZ. SI Table 2: chemical information for identified compounds of CFZ+GC. [file 5270508.f1.pdf]

Supplementary information

Crude Radix Aconiti Lateralis Preparata (Fuzi) with *Glycyrrhiza* Reduces Inflammation and Ventricular Remodeling in Mice through TLR4/NF-kB Pathway

Ping Yan [1#], Wen Mao[3#], Lushuai Jin[2],Mingsun Fang[2], Xia Liu[2], Jiali Lang[2], Lu Jin[2], Beibei Cao[2], Qiyang Shou[2*], Huiying Fu [2*]

[1] Department of Internal Medicine, University Hospital, Qufu Normal University, Qufu, China

[2] The Second Clinical Medical College, Zhejiang Chinese Medical University, Hangzhou, China

[3] School of Basic Medicine, Zhejiang Chinese Medical University, Hangzhou, China

[#]These authors contributed equally to this work.

*The corresponding authors: Huiying Fu, Qiyang Shou.

E-mail: fhy131@126.com, sqy133@126.com.

SI Table 1 Chemical information for identified compounds of CFZ

Name	Formula	Mass	RT	Score	Area
Adenine	$C_5H_5N_5$	135.0546	3.002	99.55	481590
p-hydroxylphenylpropanol	$C_9H_{10}O_3$	166.0633	5.613	99.48	586522
Dimethyl phthalate	$C_{10}H_{10}O_4$	194.0584	4.272	97.96	378158
D(-)-Lyxose	$C_5H_{10}O_5$	150.0534	1.052	97.81	470771
Benzoylmesaconine	$C_{31}H_{43}NO_{10}$	589.2893	7.874	97.67	79389
Myristic acid	$C_{14}H_{28}O_2$	228.2097	18.14	96.87	1183470
Shikimic acid	$C_7H_{10}O_5$	174.0536	1.625	96.83	16365449
Benzoylhypaconine	$C_{31}H_{43}NO_9$	573.2945	8.606	96.6	54656
Theobromine	$C_7H_8N_4O_2$	180.0648	1.06	96.58	8714017
Cholic acid	$C_{24}H_40O_5$	408.2887	10.21	95.77	95901
Gentiopicrin	$C_{16}\ H_2O_9$	356.112	4.258	94.89	398968
Sucrose	$C_{12}H_{22}O_{11}$	342.1174	1.085	94.7	253910
Nystose	$C_{24}H_{42}O_{21}$	666.2225	0.986	91.87	61577
Isoscopoletin	$C_{10}H_8O_4$	192.0412	7.524	90.98	132799
D-(+)-Raffinose	$C_{18}H_{32}O_{16}$	504.1706	1.028	88.38	145695
Sorbic acid	$C_6H_8O_2$	112.0524	1.629	87.27	556878
L-Lysine	$C_6H_{14}N_2O_2$	146.1059	1.003	87.11	59632
4'-Hydroxyacetophenone	$C_8H_8O_2$	136.0526	7.782	86.96	531822
5-Hydroxymethyl-2-Furaldehyde	$C_6H_6O_3$	126.0321	1.335	86.55	21766
γ-Aminobutyric acid	$C_4H_9NO_2$	103.0637	1.065	86.47	50679
α-L-Rhamnose	$C_6H_{12}O_5$	164.0697	1.038	86.34	111433
4-Hydroxybenzoic acid	$C_7H_6O_3$	138.0321	5.064	86.19	297038

3,4-Dimethoxybenzoic acid	$C_9H_{10}O_4$	182.0583	4.842	86.05	132271
Fumaric acid	$C_4H_4O_4$	116.0112	1.078	86.03	25153
L-Phenylalanine	$C_9H_{11}NO_2$	165.0795	5.29	85.87	40745
L-Tryptophan	$C_{11}H_{12}N_2O_2$	204.0904	3.902	85.84	160520
L(+)-Arginine	$C_6H_{14}N_4O_2$	174.1123	1.387	85.53	216582
Curcumenol	$C_{15}H_{22}O_2$	234.1625	15.411	85.48	136484
Caffeic acid	$C_9H_8O_4$	180.0429	6.984	85.47	467638
L-Histidine	$C_6H_9N_3O_2$	155.0701	1.045	85.44	190423
(±)-Camphor	$C_{10}H_{16}O$	152.1204	12.938	85.38	38821
p-Coumaric acid	$C_9H_8O_3$	164.0478	4.607	85.26	50954
Sclareol Glycol	$C_{16}H_{3}0O_2$	254.2253	18.719	84.94	693013
Fructo-oligosaccharide DP7/GF6	$C_{42}H_{72}O_{36}$	1152.3766	0.98	84.65	13683
6-Gingerol	$C_{17}H_{26}O_4$	294.1838	11.793	84.02	36383
2-Chromanone	$C_9H_8O_2$	148.052	7.547	83.83	36085
Linoleic acid	$C_{18}H_{32}O_2$	280.2411	19.02	83.75	311995
Salidroside	$C_{14}H_{2}0O_7$	300.1216	4.876	83.43	9723
Butyl p-hydroxybenzoate	$C_{11}H_{14}O_3$	194.0941	11.435	83.29	52332
Methyl 4-hydroxycinnamate	$C_{10}H_{10}O_3$	178.0624	3.13	83.17	52435
Dibutyl phthalate	$C_{16}H_{22}O_4$	278.1522	12.492	82.64	10731
Isofraxidin	$C_{11}H_{10}O_5$	222.0527	1.604	81.95	40658
12-Epinapelline	$C_{22}H_{33}NO_3$	359.2469	5.889	80.65	41365
Pectolinarigenin	$C_{17}H_{14}O_6$	314.0802	9.939	80.35	27408
Ophiopogonanone C	$C_{19}H_{16}O_7$	356.0903	8.74	80.25	19555
Bilobalide	$C_{15}H_{18}O_8$	326.1012	4.533	80.07	14701

SI Table 2 Chemical information for identified compounds of CFZ+GC

Name	Formula	Mass	RT	Score	Area
p-hydroxylphenylpropanol	$C_9H_{10}O_3$	166.0631	5.613	99.49	421094
Benzoylmesaconine	$C_{31}H_{43}NO_{10}$	589.2891	7.874	98.6	48419
p-Coumaric acid	$C_9H_8O_3$	164.0478	2.435	97.9	385401
D(-)-Lyxose	$C_5H_{10}O_5$	150.0534	1.052	97.89	489626
Isoliquiritin	$C_{21}H_{22}O_9$	418.1273	5.607	97.73	73666
Adenine	$C_5H_5N_5$	135.0545	3.002	97.65	317450
Myristic acid	$C_{14}H_{28}O_2$	228.2096	18.14	97.58	614712
Shikimic acid	$C_7H_{10}O_5$	174.0535	1.625	97.54	16679464
Cholic acid	$C_{24}H_{40}O_5$	408.2884	10.21	97.38	99962
Engeletin	$C_{21}H_{22}O_{10}$	434.1223	7.648	97.11	103492
Dimethyl phthalate	$C_{10}H_{10}O_4$	194.0586	4.272	96.97	227642
Dihydrodaidzein	$C_{15}H_{12}O_4$	256.0744	6.938	96.84	648056
Theobromine	$C_7H_8N_4O_2$	180.0648	1.06	96.55	8721984
Sorbic acid	$C_6H_8O_2$	112.0524	1.629	95.88	571782

Benzoylhypaconine	$C_{31}H_{43}NO_9$	573.2944	8.606	95.57	22557
Gentiopicrin	$C_{16}H_{20}O_9$	356.112	4.258	95.03	254337
Sucrose	$C_{12}H_{22}O_{11}$	342.1175	1.085	94.55	222126
Nystose	$C_{24}H_{42}O_{21}$	666.2228	0.986	93.03	46180
Isofraxidin	$C_{11}H_{10}O_5$	222.0526	1.604	91.51	41913
8-Methylretusin-7-O-glucopyranoside	$C_{23}H_{24}O_{10}$	460.1381	8.147	91.43	44788
Buddleoside	$C_{28}H_{32}O_{14}$	592.1812	7.986	90.58	37211
D-(+)-Raffinose	$C_{18}H_{32}O_{16}$	504.1703	1.028	90.39	244604
α-L-Rhamnose	$C_6H_{12}O_5$	164.0697	1.038	88.55	83129
Liguirtigenin-7-O-D-apiosyl-4'-O-D-Glucoside	$C_{26}H_{30}O_{13}$	550.171	5.674	88.49	63723
4'-Hydroxyacetophenone	$C_8H_8O_2$	136.0526	7.782	87.41	538532
L-Tryptophan	$C_{11}H_{12}N_2O_2$	204.0903	3.902	86.37	100963
L-Histidine	$C_6H_9N_3O_2$	155.07	1.045	86.25	114543
Butyl p-hydroxybenzoate	$C_{11}H_{14}O_3$	194.0942	11.435	86.07	47361
5-Hydroxymethyl-2-Furaldehyde	$C_6H_6O_3$	126.0322	1.335	86.07	16675
Caffeic acid	$C_9H_8O_4$	180.0428	6.984	86.01	252536
L-Lysine	$C_6H_{14}N_2O_2$	146.1059	1.003	85.81	33357
3,4-Dimethoxybenzoic acid	$C_9H_{10}O_4$	182.0583	4.842	85.61	79501
Curcumenol	$C_{15}H_{22}O_2$	234.1626	15.411	85.52	132335
Salidroside	$C_{14}H_{20}O_7$	300.1213	4.876	85.03	14733
L-Phenylalanine	$C_9H_{11}NO_2$	165.0794	5.29	84.99	27107
(±)-Camphor	$C_{10}H_{16}O$	152.1207	12.938	84.65	35483
Sclareol Glycol	$C_{16}H_{30}O_2$	254.2254	18.719	84.3	511025
Linoleic acid	$C_{18}H_{32}O_2$	280.241	19.02	83.55	294345
Formononetin	$C_{16}H_{12}O_4$	268.0745	8.261	83.03	113556
6-Gingerol	$C_{17}H_{26}O_4$	294.1839	11.793	82.95	31749
Fumaric acid	$C_4H_4O_4$	116.0115	1.078	82.91	30631
Pinobanksin	$C_{15}H_{12}O_5$	272.0692	7.653	82.41	36677
γ-Aminobutyric acid	$C_4H_9NO_2$	103.0636	1.065	82.39	71610
L(+)-Arginine	$C_6H_{14}N_4O_2$	174.1122	1.387	82.38	37903
Bilobalide	$C_{15}H_{18}O_8$	326.1011	3.346	81.99	39793
Ophiopogonanone C	$C_{19}H_{16}O_7$	356.0902	8.74	81.98	15359
Methyl gallate	$C_8H_8O_5$	184.0373	1.632	81.32	42937
Tetrahydrojateorrhizine	$C_{20}H_{23}NO_4$	341.1638	5.52	81.15	18445
Inosine	$C_{10}H_{12}N_4O_5$	268.0806	1.067	80.98	91172
